# Clinicopathological characteristics of high microsatellite instability/mismatch repair-deficient colorectal cancer: A narrative review

**DOI:** 10.3389/fimmu.2022.1019582

**Published:** 2022-12-23

**Authors:** Wei-Jian Mei, Mi Mi, Jing Qian, Nan Xiao, Ying Yuan, Pei-Rong Ding

**Affiliations:** ^1^ Department of Colorectal Surgery, State Key Laboratory of Oncology in South China, Collaborative Innovation Center for Cancer Medicine, Sun Yat-sen University Cancer Center, Sun Yat-sen University, Guangzhou, China; ^2^ Department of Medical Oncology (Key Laboratory of Cancer Prevention and Intervention, China National Ministry of Education, Key Laboratory of Molecular Biology in Medical Sciences), The Second Affiliated Hospital, Zhejiang University School of Medicine, Hangzhou, China; ^3^ Global Medical Affairs, MSD China, Shanghai, China; ^4^ Zhejiang Provincial Clinical Research Center for CANCER, Hangzhou, China; ^5^ Cancer Center of Zhejiang University, Hangzhou, China

**Keywords:** colorectal cancer, high microsatellite instability, deficient mismatch repair, microsatellite instability, characteristics, prognosis, therapy development, review

## Abstract

Colorectal cancers (CRCs) with high microsatellite instability (MSI-H) and deficient mismatch repair (dMMR) show molecular and clinicopathological characteristics that differ from those of proficient mismatch repair/microsatellite stable CRCs. Despite the importance of MSI-H/dMMR status in clinical decision making, the testing rates for MSI and MMR in clinical practice remain low, even in high-risk populations. Additionally, the real-world prevalence of MSI-H/dMMR CRC may be lower than that reported in the literature. Insufficient MSI and MMR testing fails to identify patients with MSI-H/dMMR CRC, who could benefit from immunotherapy. In this article, we describe the current knowledge of the clinicopathological features, molecular landscape, and radiomic characteristics of MSI-H/dMMR CRCs. A better understanding of the importance of MMR/MSI status in the clinical characteristics and prognosis of CRC may help increase the rates of MMR/MSI testing and guide the development of more effective therapies based on the unique features of these tumors.

## 1 Introduction

Colorectal cancers (CRCs) with high microsatellite instability (MSI-H) and deficient mismatch repair (dMMR) are a unique subgroup of cancers of the colon and rectum. The molecular and clinicopathological characteristics of MSI-H/dMMR CRCs are distinct from those of proficient mismatch repair (pMMR)/microsatellite stable (MSS) CRCs (1).

Because of their unique etiology and clinicopathological characteristics, MSI-H/dMMR and pMMR/MSS CRCs respond differently to treatment (2). This is particularly true for immune checkpoint inhibition, as MSI-H/dMMR CRCs are more immunogenic and show a better response to immunotherapy than pMMR/MSS CRCs (3, 4). Recent clinical studies showed that, in patients with advanced or metastatic MSI-H/dMMR CRC, pembrolizumab treatment led to an objective response rate (ORR) of 40.0% (95% confidence interval [CI], 12.0–74.0) (5) and the ORR in patients treated with the combination of ipilimumab and nivolumab was 54.6% (95% CI, 45.2–63.8) (6). In addition, pembrolizumab led to a significantly longer progression-free survival (PFS) than chemotherapy when administered as first-line therapy for metastatic MSI-H/dMMR CRC (hazard ratio [HR] for disease progression or death, 0.10; *P* < 0.001), with fewer treatment-related adverse events (5). KEYNOTE-177, a phase 3 study of 307 previously untreated patients with metastatic MSI-H/dMMR CRC showed that first-line pembrolizumab was superior to chemotherapy in improving PFS (HR for progression, 0.60; 95% CI, 0.45–0.80; *P* = 0.0002) and ORR (43.8% [95% CI, 35.8–52.0] *vs*. 33.1% [95% CI, 25.8–41.1]) (7). The identification of MSI-H/dMMR as a potential biomarker for response to immunotherapy in patients with CRC has led to the initiation of various clinical trials evaluating the use of neoadjuvant immunotherapy in patients with early-stage disease. Preliminary findings from the exploratory NICHE study (NCT03026140) suggest that neoadjuvant immunotherapy with nivolumab plus ipilimumab may be a suitable regimen for patients with dMMR early-stage colon cancer (8). The ability of neoadjuvant treatment with nivolumab plus ipilimumab to improve outcomes was confirmed in patients with locally advanced dMMR colon cancer (9). Furthermore, neoadjuvant treatment with immunotherapy (nivolumab plus ipilimumab) in combination with the COX-2 inhibitor celecoxib in patients with non-metastatic dMMR CRC led to a major pathologic response in 97% of patients (95% CI, 91–100; 31 of 32) (10). The efficacy of immunotherapy in combination with other treatments (e.g., chemotherapy and radiotherapy) is also being investigated in multiple ongoing trials, including VOLTAGE-A (NCT02948348), AVANA (NCT03854799), NRG-GI002 (NCT02921256), and PANDORA (NCT04083365) (11–14). Neoadjuvant immunotherapy alone or in combination with other therapies may provide new treatment options for patients with early-stage CRC, especially in MSI-H/dMMR CRC.

Despite the importance of MSI-H/dMMR and pMMR/MSS status in clinical decision making, the rates of microsatellite instability (MSI) and mismatch repair (MMR) testing in clinical practice remain low, even in high-risk populations (15, 16). Consequently, the real-world prevalence of MSI-H/dMMR CRC may be higher than that reported in the literature. Insufficient MSI and MMR testing leads to failure to identify patients with MSI-H/dMMR CRC who could benefit from immunotherapy (5, 6). Additionally, because of differences in the epidemiological, molecular, anatomical, and histological characteristics of MSI-H/dMMR and pMMR/MSS CRCs, failure to distinguish between these subgroups may lead to discrepancies in CRC diagnostic and prognostic features (1, 17).

In this article, we comprehensively review the current knowledge of the clinicopathological characteristics, molecular landscape, and radiological findings of MSI-H/dMMR tumors among patients with CRC. This overview of the role of MMR and MSI status in CRCs could increase the understanding of MSI-H/dMMR CRCs, help clinicians identify this subgroup of patients using available approaches besides MSI/MMR testing, and guide the development of more effective therapies based on the unique molecular characteristics of these tumors.

## 2 Molecular mechanisms of MSI-H/dMMR in CRC

Inactivation of an MMR gene by mutation or transcriptional silencing results in deficient function of the MMR system, leading to the accumulation of errors during DNA replication (18). Multiple proteins that mediate DNA repair are involved in the MMR pathway, including the MutS family (MSH2, MSH3, and MSH6) and the MutL family (MLH1, MLH3, PMS1, and PMS2). Among these proteins, MLH1, MSH2, MSH6, and PMS2 are the most important regulators of MMR (18).

**Figure 1:**
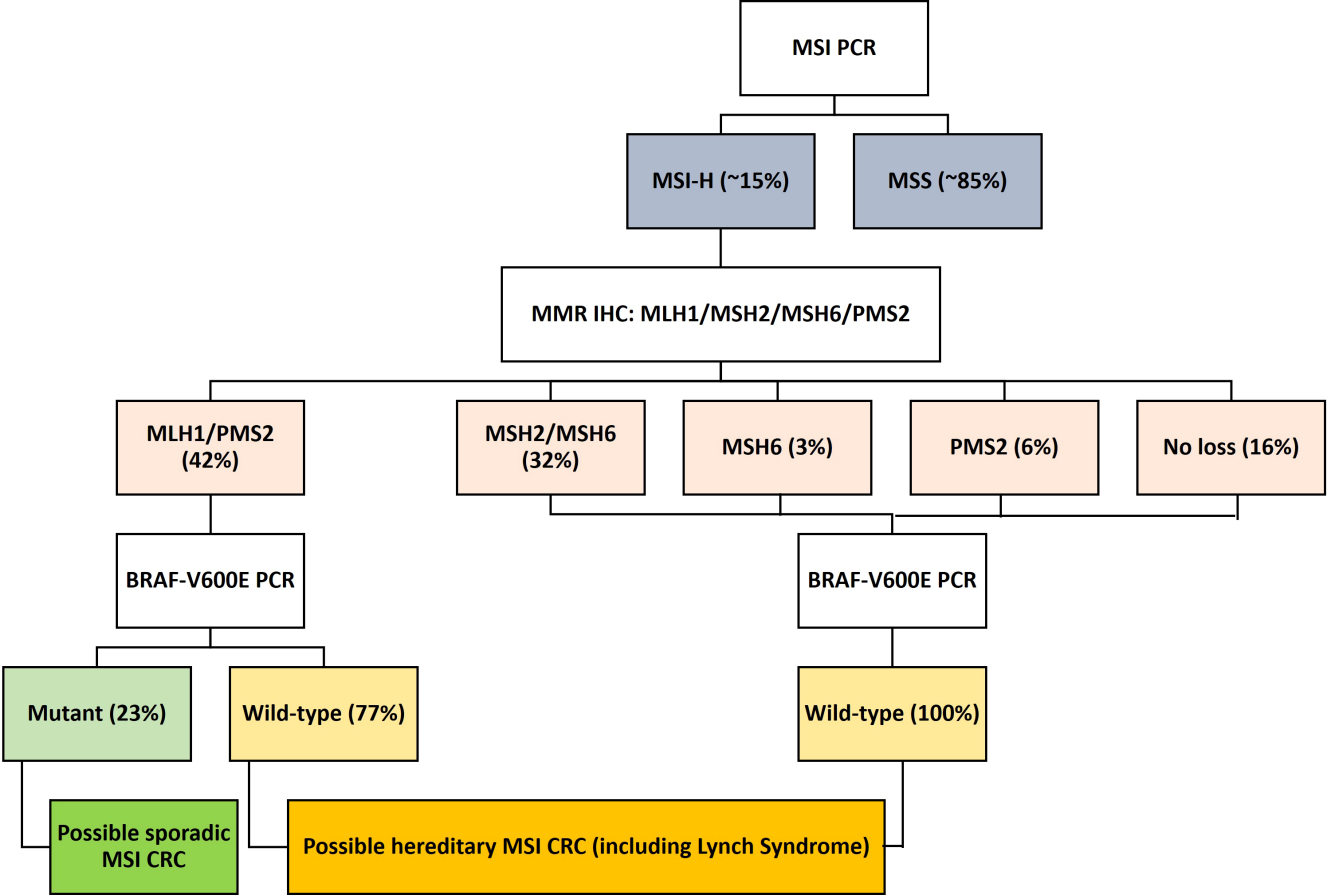
Flow diagram showing the molecular classification and frequency of sporadic and hereditary MSI-H/dMMR CRC. CRC, colorectal cancer; dMMR, deficient mismatch repair; MMR, mismatch repair; MSI-H, microsatellite instability-high.

Studies have identified two distinct molecular pathways comprising germline or somatic mutations that contribute to the inactivation of MMR genes. Germline mutations in an MMR gene followed by a second hit to the wild-type copy due to point mutations, loss of heterozygosity (LOH), or methylation (18) can inactivate the gene. Inherited colorectal syndromes contribute to the development of approximately 5% of all CRCs, of which Lynch syndrome is the most common (**Figure 1**) (18). Mutations in *MLH1* and *MSH2* are found in approximately 70% of patients with Lynch syndrome, whereas mutations in *MSH6* and *PMS2* are less common and are found in only 15% of patients (19).

DNA methylation, also referred to as CpG island methylator phenotype (CIMP), occurs in 20% of CRCs and results in a non-familial form of MSI (19). Such DNA hypermethylation results in gene silencing in most cases (19) or upregulation under certain circumstances (20, 21). Sporadic CRCs are mainly due to loss of *MLH1* expression caused by hypermethylation of the *MLH1* promoter in a CIMP background (19).

## 3 Clinical characteristics of MSI-H/dMMR CRCs

### 3.1 Demographic characteristics and MSI/MMR status in CRCs

Findings from multiple studies suggest that dMMR status is associated with early onset disease among patients with CRC, as dMMR CRCs are more frequent in younger patients than in older patients. A retrospective analysis of 133 patients with CRC showed that mutations in *MLH1*, *MSH2*, *MSH6*, and *PMS2* were significantly associated with age (22). A subsequent retrospective study of 61 patients with stage I–III CRC confirmed a significant association between dMMR status and patient age (23). A recent real-world study revealed that, among patients with dMMR CRC, dMMR tumors were observed in both older (≥60 years) and younger (<50 years) patients. The frequency of MSH6/MSH2, MSH6, and PMS2 loss was higher in younger patients than in older patients. However, the statistical significance of this finding could not be determined because the expected expression values were low in >20% of the cells (24). Among patients with Lynch syndrome, the median age at CRC diagnosis was ten years higher for carriers of *MSH6* mutations than for those carrying *MLH1* and *MSH2* mutations (25).

Similar associations have been reported for dMMR status and sex; in most studies, the percentage of women in the dMMR CRC group was higher than the percentage of men. For example, a large-scale study of 535 patients with CRC showed that tumors from women had a higher frequency of MLH1/PMS2 loss than tumors from men (26). Consistently, Viñal et al. (27) reported that the percentage of women was significantly higher among patients with dMMR CRC than among those with pMMR CRC (55% [n = 55/100] *vs*. 38% [n = 351/914]; *P* = 0.001).

### 3.2 Tumor characteristics and MSI/dMMR status in CRC

MSI-H/dMMR status has been associated with various CRC tumor characteristics, including the location of the primary tumor, tumor diameter, T stage, and distant metastasis. Several retrospective studies have shown a significant association between dMMR/MSI-H status and early onset disease, maximum tumor diameter, large tumor volume, primary tumor site, and advanced T stage in patients with stage (including tumor, node, metastasis [TNM] stage) I–III or I–IV CRC (23, 27–29).

A retrospective study of 245 patients with CRC showed that the incidence of MSI-H was higher in patients with right colon cancer and TNM stage I–II disease (30). Another retrospective analysis of 268 patients with CRC showed a high incidence of dMMR in patients with locally advanced (T4b) tumors without distant metastasis (31). Additionally, a recent analysis of 1,014 patients with CRC (100 [9.8%] with dMMR and 914 [90.2%] with pMMR tumors) indicated that advanced-stage tumors were significantly more common among patients with pMMR CRC than among those with dMMR CRC (stage IV: 21% *vs*. 3%; *P* < 0.001) (27). Similarly, Kang et al. (29) found a significant association between MSI-H and earlier-stage tumors in patients with CRC. These findings suggest that dMMR may play a protective role in CRC.

In a retrospective case series, Li et al. found that mutations in *MLH1*, *MSH2*, and *MSH6* were significantly associated with primary tumor location among patients with dMMR CRC; hMLH1 or PMS2 loss was more common on the right side, whereas hMSH2 or hMSH6 loss was more common on the left side (22). Similarly, a retrospective analysis of 795 patients found that proximal lesions were a predictor for MSI, with a multivariate odds ratio (OR [95% CI]) of 0.419 (0.223–0.784; *P* = 0.007) (32).

However, Yan et al. found that larger tumor size was associated with MSI (OR [95% CI], 1.300 [1.076–1.572]; *P* = 0.007), as did Liang et al. (median diameters, 6.0 cm in the dMMR group compared with 4.5 cm in the pMMR group; *P* < 0.01) (23, 32).

### 3.3 Histopathological and pathomorphological characteristics of MSI-H/dMMR CRCs

**Figure 2:**
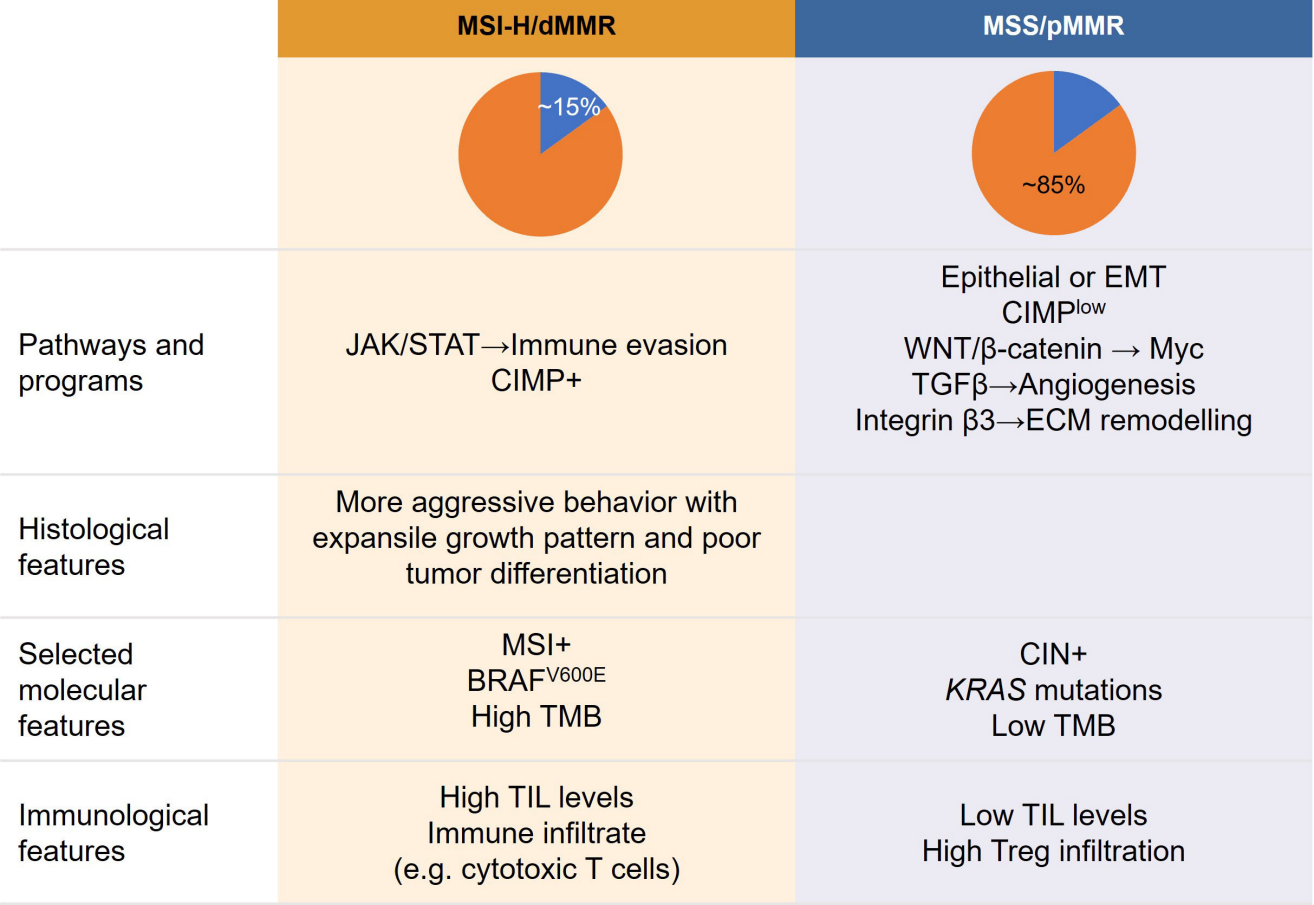
Proposed relationship between tumor features, molecular profiles, clinicopathological characteristics, and immunological features of colorectal cancer according to MSI subtype. CIMP, CpG island methylator phenotype; CIN, chromosomal instability; dMMR, mismatch repair deficient; ECM, extracellular matrix; EMT, epithelial–mesenchymal transition; JAK/STAT, Janus kinase/signal transducer and activator of transcription; MSI, microsatellite instability; MSS, microsatellite stable; pMMR, MMR proficient; TGF, transforming growth factor; TIL, tumor-infiltrating lymphocyte; TMB, tumor mutational burden; Tregs, T-regulatory cells; VEGF, vascular endothelial growth factor.

MSI-H/dMMR CRCs and pMMR/MSS/MSI-L CRCs differ in their histopathological and pathomorphological characteristics. For instance, in a study of 312 patients with colorectal adenocarcinomas, mucinous adenocarcinomas were more common among patients with dMMR CRC than among those with pMMR CRC (33). Most dMMR CRCs show aggressive histological features, including an expansile growth pattern, a high degree of tumor cell infiltration, poor tumor differentiation, and a medullary pattern (34), as summarized in **Figure 2**. Consistently, Liang et al. (23) reported a significantly higher frequency of poorly differentiated tumors in patients with dMMR CRC than in those with pMMR CRC (41.0% [n = 25/61] *vs*. 10.9% [n = 20/183]; *P* < 0.05), although no significant differences in the rates of lymphovascular invasion and extranodal extension were observed. In contrast, localized disease at diagnosis (97% *vs*. 79%; *P* < 0.001) and histological grade 3 (20% *vs*. 8%; *P* < 0.001) were more frequent in patients with dMMR CRC than in those with pMMR CRC (27).

While both serrated and non-serrated sporadic colorectal adenocarcinomas can present MSI-H (35), studies have shown that MSI is more common in conventional carcinomas than in colorectal serrated adenocarcinomas (36). Other histological and morphological features of dMMR/MSI-H CRCs include high numbers of tumor-infiltrating lymphocytes, Crohn’s-like inflammatory reaction, mucinous/focal signet ring cell differentiation, and lack of dirty necrosis within the tumor lumen (37–39).

MSI-H/dMMR status in patients with CRC has also been correlated with high infiltration levels of immune cells, including T helper 1 (Th1) cells and cytotoxic T cells, which may explain the favorable response to immunotherapy among patients with MSI-H/dMMR tumors. The high degree of immune cell infiltration in dMMR CRCs may be attributed to the high mutational burden and neoantigen load of these tumors (3, 40), making dMMR/MSI-H CRCs amenable to immunotherapy. Despite the durable responses observed in some patients with MSI-H/dMMR CRC treated with immune checkpoint inhibitors, many CRCs are resistant to immunotherapy (41, 42). High intratumoral heterogeneity due to the high rate of mutations in MSI-H/dMMR CRCs may contribute to the generation of immune escape clones, leading to the development of immunotherapy resistance (41, 43). Although tumor mutational burden (TMB) and the expression levels of PD-1/PD-L1 have been proposed as determinants of differential responses to anti–PD-1/PD-L1 treatment among CRC patients with different MSI statuses (44), further studies are required to determine the roles of PD-1/PD-L1, *BRAF*/*RAS* mutations, TMB, and T-cell phenotype as biomarkers of response to immune checkpoint inhibitors in patients with MSI-H/dMMR CRC.

Despite the significant association between dMMR status and certain clinicopathological and tumor histologic characteristics as mentioned above, the role of MSI and MMR in clinicopathological features is complex and may be confounded by multiple factors. The higher prevalence of dMMR in earlier-stage tumors indicates a protective role of dMMR, while dMMR CRCs show aggressive histological features, including an expansile growth pattern, a high degree of tumor cell infiltration, poor tumor differentiation, and a medullary pattern.

Several factors may contribute to these paradoxical observations regarding the relationship between MSI and tumor characteristics in CRC. Most studies evaluating the relationship between MSI and tumor or clinicopathological features in patients with CRC included small cohorts because dMMR CRC is relatively rare. Additionally, there are no widely established criteria for the diagnosis of MSI-H/dMMR tumors. Variations in the evaluation of MMR status may contribute to contradictory findings regarding the predictive and prognostic roles of MSI-H/dMMR status in CRC. There is also evidence to suggest heterogeneous characteristics among the different subgroups of MSI-H/dMMR CRCs. For example, hereditary and sporadic MSI-H CRCs differ in their histological and morphological characteristics (45). Substantial racial differences in the tumor microenvironment of CRCs have also been reported (46). Moreover, because of defects in DNA repair pathways, dMMR CRCs have substantial genetic instability, which could lead to intertumoral molecular heterogeneity (17, 47). Accumulation of genetic mutations during the progression of MSI-H/dMMR CRCs can also lead to the acquisition of more aggressive features. These and other confounding factors must be accounted for in studies evaluating the role of MSI in the characteristics of CRC.

### 3.4 Pathomics and artificial-intelligence–assisted prediction of MSI-H/dMMR status in CRC

Advances in artificial intelligence (AI) have augmented the development of pathomics and AI-assisted methods for the characterization of tumors from patients with CRC. Most research efforts have focused on the development of models to automate the analysis and increase the accuracy of gland segmentation, tumor classification, tumor microenvironment characterization, and prognostication (48, 49).

Significant progress has also been made in the development of AI-assisted models to predict MSI status in CRC based on the distinct histomorphological features of MSI-H/dMMR tumors (**Table 1**) (61). For example, an open-source AI system that was trained using routine pathology slides from eight multicenter cohorts facilitated accurate and fully automated prediction of MSI status, yielding an area under the curve (AUC) value of 0.96 (95% CI, 0.94–0.98) (50). The system was successfully applied as a rule-out test to predict MSS/pMMR and identify patients with CRC for whom molecular MSI testing is not required. Similarly, Cao et al. (51) developed a pathomics-based deep-learning model trained using histological data from The Cancer Genome Atlas (TCGA) and an Asian CRC cohort. The model accurately predicted MSI status from histopathological images, with an AUC of 0.8848 (95% CI, 0.8185–0.9512) in the TCGA cohort and 0.8504 (95% CI, 0.7591–0.9323) in the Asian CRC cohort. The model accurately captured various characteristics of MSI-H tumors, including poor differentiation and high TMB (51). In an effort to improve the performance of AI algorithms in predicting MSI, Saillard et al. (52) developed a self-supervised deep-learning model that was trained using histology images from the TCGA dataset. The model predicted MSI status in CRC with high sensitivity and specificity, achieving an AUC of 0.92 (95% CI, 0.84–0.99) and outperforming previous supervised deep-learning models.

**Table 1:** Emerging non-invasive methods for predicting MSI-H/dMMR status in CRC.

Method	Application	AUC (95% CI)	Reference
AI-based MSI/dMMR detector trained using routine pathology slides from eight multicenter cohorts	Rule-out test for predicting MSS/pMMR and identifying patients with CRC for whom molecular MSI testing is not required	0.96 (0.94–0.98)	(50)
Pathomics-based deep learning model trained using histological data from TCGA and an Asian CRC cohort	Prediction of MSI status from histopathology images	TCGA cohort: 0.8848 (0.8185–0.9512) Asian CRC cohort: 0.8504 (0.7591–0.9323)	(51)
Self-supervised deep learning model that was trained using histology images from TCGA	Prediction of MSI status from histology slides	0.92 (0.84–0.99)	(52)
Convolutional neural network-based algorithm	Prediction of TMB-H status (defined as MSI-H, high TMB, or both) from H&E-stained slides of CRC tissues	0.934 (0.835–0.981	(53)
Serum CEA levels	Prediction of dMMR status in patients with CRC	0.546	(54)
Serum CA 72-4 levels	Prediction of dMMR status in patients with CRC	0.583	(54)
Combination of serum levels of CA 72-4 and CEA with patient age, histology type, tumor size, tumor location, degree of differentiation, LN metastasis, and peripheral nerve invasion	Prediction of dMMR status in patients with CRC	0.849	(54)
Age, tumor diameters, histology, tumor location, perineural invasion, the number of sampled LNs and positive LNs	Prediction of dMMR status in patients with CRC	Primary cohort: 0.756 (0.722–0.789 Validation cohort: 0.754 (0.715–0.793	(55)
Combination of serum levels of CA 72-4 and CEA with age, tumor diameters, histology, tumor location, perineural invasion, the number of sampled LNs, and positive LNs	Prediction of dMMR status in patients with CRC	Primary cohort: 0.805 (0.774–0.835) Validation cohort: 0.796 (0.758–0.835)	(55)
CT-based nomogram consisting of six radiomic features and 11 clinical characteristics	Prediction of MSI status in patients with stage II CRC	0.752	(56)
Preoperative triphasic enhanced CT radiomics signatures consisting of 32 features	Prediction of MSI status in patients with CRC	Primary cohort: 0.898 (0.860–0.937) Validation cohort: 0.964 (0.919–1.000)	(57)
Nomogram integrating clinical, pathological, and radiomics data	Prediction of MSI status in patients with rectal cancer	0.757 (0.726–0.787	(58)
Machine learning model trained using both tumoral and peritumoral radiomic signatures	Prediction of MSI status in patients with rectal cancer	Primary cohort: 0.817 (0.772–0.856) Validation cohort: 0.726 (0.648–0.796)	(59)
Model integrating six MRI-derived radiomic features and clinical characteristics	Preoperative prediction of MSI status in patients with rectal cancer	0.895 (0.838–0.938	(60)

AI, artificial intelligence; AUC, area under the curve; CEA, carcinoembryonic antigen; CRC, colorectal cancer; CT, computed tomography; H&E, hematoxylin and eosin; LN, lymph node; MRI, magnetic resonance imaging; MSI-H/dMMR CRC, microsatellite instability-high/deficient mismatch repair colorectal cancer; MSI, microsatellite instability; MSS, microsatellite stable; pMMR, mismatch repair proficient; TCGA, The Cancer Genome Atlas.

AI-assisted algorithms have also been developed to predict TMB-H status, which is strongly correlated with MSI-H/dMMR status. Shimada et al. (53) developed a convolutional neural network-based algorithm to predict TMB-H status (defined as MSI-H, high TMB, or both) from hematoxylin and eosin (H&E)-stained slides of CRC tissues. The model integrated various histomorphological features of MSI-H/dMMR CRCs, including increased lymphocytic infiltration, abundance of peritumoral lymphocytes, mucinous features, Crohn’s-like inflammatory reaction, and medullary features. The model accurately predicted TMB-H status, providing an AUC of 0.934 (range, 0.835–0.981) (53).

Current testing strategies for MSI-H/dMMR status include polymerase chain reaction (PCR), next-generation sequencing (NGS), and immunohistochemistry (IHC). High testing costs and limited resources are critical factors that hinder the wider application of MSI testing in patients with CRC. Kacew et al. (62) used a representative population-based sample of individuals receiving first-line treatment for metastatic CRC (N = 32,549) in the US to estimate the clinical and financial consequences of predicting MSI status in CRC using AI-assisted methods instead of conventional methods. Their model showed that, compared with current testing strategies, MSI testing using AI followed by confirmatory PCR or IHC testing for patients testing dMMR/MSI-H-positive by AI resulted in the lowest population-level diagnostic costs (including testing and first-line drug costs) in this cohort ($400 million [12.9%] lower than that of NGS alone). The method also maintained 91% diagnostic accuracy and facilitated timely diagnosis (62). However, a key limitation of this study was that population-based costs were estimated using a model, and no real-world validation of costs was conducted. Hence, it is possible that AI-related costs were underestimated, as additional costs of applying AI in real-world clinical settings (e.g., internal validation, maintenance of hardware and software, and scanning slides) were not taken into account. Further validation of AI-assisted MSI prediction algorithms in large datasets and real-world cohorts is required to support the clinical adoption of these models in routine practice.

### 3.5 Molecular characteristics and MSI/MMR status in CRCs

MSI-H tumors have high genetic instability, and dMMR/MSI-H CRCs exhibit extensive intratumoral and intertumoral molecular heterogeneity (47). A key feature of MSI-H CRCs is the lack of MMR proteins or deletions in MMR-related genes. Hence, MSI-H/dMMR CRCs can be distinguished from MSS/pMMR CRCs using IHC, PCR, or NGS. Epigenetic mechanisms (e.g., DNA methylation) also contribute to the loss of MMR proteins in dMMR/MSI-H CRC. Thus, numerous methods have been developed to detect epigenetic alterations in clinical samples from patients with CRC.

The frequencies of *TP53* loss, *MLH1* promoter methylation, and *KRAS* and *BRAF* mutations vary between MSI-H and MSS CRCs (**Table 2**) (1, 63). *BRAF*-V600E mutations are more common in MSI-H CRCs than in MSS CRCs. In contrast, *KRAS* mutations and *TP53* loss are more frequent in MSS than in MSI-H CRCs (1, 63). Approximately 80% of dMMR CRCs exhibit *MLH1* promoter methylation (67). Because most sporadic dMMR CRCs exhibit *MLH1* promoter methylation and many also have *BRAF* mutations, *MLH1* promoter methylation analysis can be used to discriminate sporadic tumors from Lynch syndrome in patients without *BRAF* mutations. Absence of *BRAF* mutations and *MLH1* promoter methylation in tumors is associated with hereditary forms of CRC (67, 68). In contrast, the *BRAF*-V600E mutation in patients with dMMR CRC is strongly associated with sporadic tumors (69). Several clinicopathological characteristics, including age at diagnosis, tumor location, and patient survival, differ between patients with MLH1-deficient/*BRAF*-V600E–mutated dMMR CRC and those with MLH1-deficient/*BRAF* wild-type dMMR CRC (70, 71). Interestingly, the characteristics of *BRAF*-mutated MSS CRCs appear to be distinct from those of *BRAF*-mutated MSI-H CRCs and *BRAF* wild-type MSS CRCs. Landau et al. analyzed 205 CRCs and found that stage IV tumors at diagnosis were significantly more common among patients with *BRAF*-mutated MSS CRCs than among those with *BRAF*-mutated MSI-H CRCs and *BRAF* wild-type MSS CRCs (*P* < 0.001) (64). They also found that cytokeratin 7 (CK7) loss was significantly more common in *BRAF*-mutated MSI-H and *BRAF* wild-type MSS CRCs than in *BRAF*-mutated MSS CRCs (*P* = 0.0001). Furthermore, cytokeratin 20 (CK20) loss was more common in *BRAF*-mutated MSI-H CRCs than in *BRAF*-mutated MSS and *BRAF* wild-type MSS CRCs (*P* = 0.001) (64). *BRAF* mutations in patients with metastatic dMMR/MSI-H CRC have been associated with poor outcomes, including shorter overall survival (OS) (72).

**Table 2:** Frequency of common mutations in MSI-H and MSS CRCs.

Genetic alteration	Mutation Frequency, %		Reference
	**MSI-H CRC**	**MSS CRC**	
*TP53* loss	31.6	46.4	(63)
*KRAS* mutations	36.8	2.3	(63)
*BRAF-*V600E mutation	36.8	1.0	(63)
Cytokeratin 7 loss	94^a^	61^a^	(64)
Cytokeratin 20 loss	30^a^	7^a^	(64)
*CTNNB1* mutations	10	0.7	(65)
*HNF1A* mutations	32	0.2	(65)
*BRCA1* mutations	19	5	(65)
*BRCA2* mutations	50	14	(65)
Thymidylate synthase upregulation	85	31	(65)
PTEN upregulation	71	48	(65)
*HER2* mutations	5.6	3.7	(66)

a Tumors harbored *BRAF* mutations.

Because MSI-H tumors are more genetically unstable than MSS tumors, MSI-H CRCs tend to accumulate mutations in various oncogenes and tumor suppressor genes, including *BRAF*, *CTNNB1*, *HNF1A*, *PTEN*, *BRCA1*, and *BRCA2* (65, 73). Consequently, MSI-H CRCs have a higher TMB and neoantigen load than MSS CRCs (66, 74). Advances in NGS have contributed to the identification of several mutations associated with dMMR/MSI-H status in CRC. NGS analysis of tissues from 430 patients with CRC showed that mutations in MAPK pathway genes (e.g., *KRAS*, *NRAS*, *BRAF*) and *HER2* were significantly more frequent in MSI-H CRCs than in MSS tumors (83.6% *vs*. 58.4%, *P* = 0.0003) (66).

In line with the high TMB of dMMR/MSI-H CRCs, NGS analysis of tumor samples from 64 patients with CRC showed that MSI-H tumors harbored a total of 1756 alterations (mean, 125; range 63–302) across 447 genes, whereas MSS tumors had only 493 alterations (mean, 10; range 1–26) across 186 genes (75). Among the total of 633 mutated genes, only 165 were altered in both groups. Both MSI-H and MSS tumors harbored mutations in *APC*, *TP53*, and *KRAS*, which are among the most frequently mutated genes in CRC. The most commonly altered genes that were mutated only in MSI-H tumors were *ANKRD11* (78.6%), *ARID1A* (71.4%), *KMT2B* (71.4%), *BCORL1* (64.3%), *IGF1R* (50.0%), *KDM5* (50.0%), *POLD1* (50.0%), and *TSC1* (50.0%). Additionally, mutations in microsatellite loci (mononucleotide repeats) were more frequent in MSI-H CRCs than in MSS tumors.

Interestingly, the serum levels of molecular tumor markers, including CEA, CA 19-9, and CA 72-4, have also been associated with MSI/MMR status in CRC (55, 63). A retrospective analysis of samples from 2279 patients with CRC indicated that dMMR status was associated with normal CEA serum levels and elevated CA 72-4 levels (54). The use of serum CEA levels to predict dMMR status yielded AUC scores of 0.546 in the entire cohort and 0.554 in the TNM II/III subgroup. Similarly, serum CA 72-4 levels showed a modest ability to predict dMMR status, with an AUC score of 0.583 (54). Although the ability of individual serum tumor markers to predict MSI/MMR status is limited, the combination of serum markers and clinicopathological features may help identify patients with MSI-H/dMMR CRC (55). The combination of serum levels of CA 72-4 and CEA with patient age, histology type, tumor size, tumor location, degree of differentiation, lymph node metastasis, and peripheral nerve invasion to predict dMMR status in patients with CRC provided an AUC score of 0.849, which was considerably higher than the AUC scores of individual markers (54). Another retrospective analysis of 3,274 patients with CRC confirmed that the addition of CEA and CA 72-4 to dMMR prediction models significantly improved the discriminative ability of a pathology-based model in the primary (AUC: 0.805 [95% CI, 0.774–0.835] *vs*. 0.756 [95% CI, 0.722–0.789]; *P <* 0.001) and validation cohorts (AUC: 0.796 [95% CI, 0.758–0.835] *vs*. 0.754 [95% CI, 0.715–0.793]; *P <* 0.001) (55).

### 3.6 Radiomic characteristics of MSI-H/dMMR CRCs

Using non-invasive methods to predict MSI status prior to treatment or surgery remains an unmet clinical need. The usefulness of traditional radiological evaluation of CRCs using computed tomography (CT) to predict MSI status is limited. Therefore, novel CT technologies and radiomic features to predict MSI/MMR status in CRC have been evaluated in several studies (**Table 1**).

In a recent radiomics analysis of iodine-based material decomposition images captured by dual-energy CT imaging, a nomogram based on a combination of clinical factors and radiomics scores predicted MSI status in pretreatment patients with CRC (76). Preliminary findings from a retrospective study indicated that a CT-based nomogram consisting of six radiomic features and 11 clinical characteristics could predict MSI status in patients with stage II CRC, yielding an AUC of 0.752 (sensitivity, 0.663; specificity, 0.842) (56). Consistently, a multicenter study demonstrated that preoperative triphasic enhanced CT radiomics signatures consisting of 32 features could predict MSI status in 502 patients with CRC (57). Delayed-phase models were superior to arterial- or venous-phase models in predicting MSI status. Although these studies demonstrated the feasibility of using radiomic features to predict MSI status in CRC, the nomograms were developed based on data from relatively small patient cohorts.

To develop an MSI-predictive nomogram based on radiomics data from a large cohort, Pei et al. (77) used texture analytical software to extract pelvic CT radiomic features from 762 patients with CRC. Patients with MSI-H tumors showed significantly higher radiomics nomogram scores, suggesting that pretreatment radiomic features can be used as a non-invasive method to predict MSI status in patients with CRC (77). In contrast, another study involving the development and validation of a model to predict MSI status by integrating clinical, pathological, and radiomics data from a large cohort of patients with rectal cancer (n = 788) showed that the nomogram provided a moderate ability to predict MSI status. However, the model provided a higher AUC than clinical, pathological, and radiomic features alone (AUCs: 0.757, 0.584, 0.585, and 0.737, respectively) (58). In contrast to previous efforts to develop MSI-prediction models based on tumoral CT-based radiomics, Ma et al. (59) developed a machine-learning model to predict MSI status using both tumoral and peritumoral radiomic signatures. The model predicted MSI status in rectal cancer, achieving AUCs of 0.817 (95% CI, 0.772–0.856) and 0.726 (95% CI, 0.648–0.796) in the training and validation sets, respectively.

Positron emission tomography (PET)/CT has also been used as a non-invasive method to predict MSI status in patients with CRC and analysis of quantitative imaging markers *via* statistical modelling may further reflect pathophysiology and allow objective evaluation of tumor heterogeneity, giving more information than a single IHC/PCR assay. Li et al. (78) conducted a radiomics analysis using preoperative ^18^FDG PET/CT images to predict MSI/MMR status in 173 patients with CRC. They identified one PET radiomic feature (wavelet-LHH_firstorder_Skewness_PET) and one CT radiomic feature (wavelet-HHL_firstorder_RootMeanSquared_CT) that were associated with MSI status (both *P* < 0.05), providing a quantitative and non-invasive approach to identify patients with MSI-H/dMMR CRC (78). Metabolic parameters derived from preoperative ^18^FDG PET/CT images have also been found to predict MSI status in 44 patients with CRC (79). Metabolic tumor volume (MTV)_30%_, MTV_40%_, MTV_50%_, MTV_60%_, total lesion glycolysis (TLG)_50%_, and TLG_60%_ differed significantly between the MSI and MSS groups (all *P* < 0.05). Among these parameters, MTV_50%_ was the strongest predictor of MSI (79). Although PET is expensive and may not be readily available in all clinics, predicting MSI status using PET/CT is non-invasive and does not require tissue biopsy. Therefore, it could be adopted for patients with insufficient biopsy tissues for IHC/PCR testing or without biopsy tissue. Even though IHC is inexpensive and widely available, variations in IHC fixation and staining protocols and objectivity in scoring may influence its accuracy in identifying dMMR/MSI-H tumors.

MRI-based radiomic features have also been used to develop non-invasive models to predict MSI status in CRC. For example, Zhang et al. (60) developed a model integrating six MRI-derived radiomic features and clinical characteristics to predict MSI preoperatively in 491 patients with rectal cancer. The combined model yielded an AUC of 0.895 (95% CI, 0.838–0.938), which was significantly higher than that obtained using clinical characteristics alone (AUC: 0.685 [95% CI, 0.608–0.755]; *P* = 0.015).

### 3.7 Racial disparities in MSI-H/dMMR CRC

Racial differences in the prevalence of MSI-H/dMMR CRC have been reported (**Table 3**) (102–104). For example, the incidence of MSI-H CRC is relatively high among Egyptians (37%), African Americans (12%–45%), Europeans (5%–24%), and Caucasian Americans of European descent (8%–20%) (73, 80, 86–88, 105). In contrast, the reported incidence of MSI-H CRC in Asian countries is relatively low, ranging from 3.8% to 20.0% in Japan (92, 93) and from 4.5% to 15.0% in China (94, 95). Furthermore, the reported frequency of dMMR in synchronous CRCs is lower in Japanese patients than in Western patients (106).

**Table 3:** Incidence of MSI-H/dMMR colorectal cancer in different populations.

Population	Incidence, %	Reference
Egyptians	37.0	(80)
Europeans	
Greece	5.0	
Romania	21.1	(81)
Germany	23.7	(82)
Scandinavia	7.0	(83)
The Netherlands	3.5	(84)
UK	22.9	(85)
African Americans	12.0–45.0	(86, 87)
Caucasian Americans	19.0	(88)
US Latino/Hispanic individuals	13.0	(89)
Mexicans	21.3–27.1	(90, 91)
Japanese	4.0–20.0	(92, 93)
Chinese	5.0–15.0	(94–96)
Koreans	16.0	(97)
Indians	1.0–29.5	(98–101)

In line with racial differences in the prevalence of MSI-H/dMMR CRC, accumulating evidence suggests that racial/ethnic disparities also exist in the genetic profiles of CRCs (102, 107, 108). Zhang et al. sequenced tumors from 1,110 Chinese patients with CRC to identify oncogenic mutations. They found that 45.4%, 3.9%, 3.1%, and 3.5% of tumors harbored mutations in *KRAS*, *NRAS*, *BRAF*, and *PIK3CA*, respectively (109). Interestingly, the frequency of the *BRAF* V600E mutation was 3.1%, which is lower than that reported in studies conducted in Western countries. To identify racial differences in the tumor microenvironment of colon cancers, Paredes et al. analyzed gene expression in tumor tissues in a US cohort. They found that tumors from African American patients had higher expression levels of *FOXP3*, *IL1B*, and *IL8* than tumors from Caucasian Americans (all *P* < 0.05) (46). In contrast, tumors from Caucasian Americans had higher expression levels of markers associated with antitumor immune responses, including *GZMB*, *IFNG*, *CD274* (encoding PD-L1), and *CTLA4* (all *P* < 0.05).

The combination of non-modifiable genetic factors (e.g., family history, genetic polymorphisms) and environmental factors (e.g., diet, body weight, sedentary lifestyle, and exercise) may contribute to racial and ethnic disparities in the incidence and mortality of CRC (109–111).

### 3.8 Disparities of MSI-H/dMMR CRCs in hereditary *vs*. sporadic CRCs

Family history, age at disease onset, and prognosis are among the clinical characteristics that differ between patients with Lynch syndrome and those with sporadic MSI-H CRC. In contrast to patients with Lynch syndrome, those with sporadic MSI-H CRC often have no family history of CRC (45). Consistent with the role of germline mutations in hereditary colorectal syndromes, Lynch syndrome typically presents earlier in life than sporadic MSI-H CRCs (45, 112). Additionally, patients with stage I–III MSI CRC have a lower OS rate than those with Lynch syndrome; however, no significant differences in recurrence-free survival rates have been reported (113).

Histological and morphological differences also exist between sporadic MSI-H CRCs and Lynch syndrome. Sporadic MSI-H CRCs typically have cytoplasmic eosinophilia, and large, round, vesicular nuclei with a prominent nucleolus. In contrast, the cytological features of Lynch syndrome are similar to those of conventional adenomas (45). Furthermore, lymphocytic infiltration, tumor-cell de-differentiation, and presence of adenomas are more common in Lynch syndrome than in sporadic MSI-H CRCs. In contrast, mucin secretion, poor tumor differentiation, high intratumoral heterogeneity, Crohn’s-like reaction, glandular serration, and the presence of serrated polyps are more frequent in sporadic MSI-H CRCs than in Lynch syndrome (45, 113, 114).

Genetic factors predisposing individuals to DNA methylation may contribute to differences in clinicopathological characteristics between hereditary and sporadic MSI-H CRCs (45). Methylation of the *MLH1* promoter and *BRAF* V600E mutations are frequently detected in sporadic MSI-H CRCs but not in patients with Lynch syndrome, suggesting that testing for *BRAF* mutations and *MLH1* promoter methylation may help differentiate Lynch syndrome from sporadic MSI-H/dMMR CRC (115–117).

## 4 Conclusions

MSI CRCs possess distinct clinicopathological and molecular characteristics compared to MSS CRCs. In recent years, the development and exploration of novel testing technologies and methods, including NGS, AI-based histological algorithms, and image-based radiomic analysis, have shown promise for further defining and identifying this unique subgroup of CRCs. Combining multiple parameters with machine learning is a promising strategy to improve the performance of predictive models. The integration of histopathological and clinicopathological characteristics may improve the identification of patients with MSI-H/dMMR CRC. Progress in testing methods and predictive models has led to a deeper understanding of the disease and has important implications for patient management. The future development and utilization of these methods may hold promise for improving patient outcomes and for the development of novel therapeutics for patients with MSI-H/dMMR CRC.

## Author contributions

All authors substantially contributed to planning, gathering, and interpreting the information or ideas used in the paper. During the process, they substantially contributed to providing suggestions for revision or critically reviewing subsequent iterations of the manuscript and ensured that questions related to the accuracy or integrity of any part of the work were appropriately investigated and resolved. Finally, they all reviewed and approved the final version of the paper.
